# Interactions between Beta-2-Glycoprotein-1 and Phospholipid Bilayer—A Molecular Dynamic Study

**DOI:** 10.3390/membranes10120396

**Published:** 2020-12-05

**Authors:** Natalia Kruszewska, Krzysztof Domino, Radosław Drelich, Wiesław Urbaniak, Aneta D. Petelska

**Affiliations:** 1 Institute of Mathematics and Physics, UTP University of Science and Technology, Kaliskiego 7, 85-796 Bydgoszcz, Poland; 2 Institute of Theoretical and Applied Informatics, Polish Academy of Sciences, Bałtycka 5, 44-100 Gliwice, Poland; kdomino@iitis.pl; 3 Faculty of Mathematics, Physics and Technical Sciences, Kazimierz Wielki University, Chodkiewicza 30, 85-867 Bydgoszcz, Poland; radeko@ukw.edu.pl (R.D.); wurban@ukw.edu.pl (W.U.); 4 Faculty of Chemistry, University of Bialystok, Ciolkowskiego 1K, 15-425 Bialystok, Poland

**Keywords:** beta-2-glycoprotein-1, DPPC, POPE, molecular dynamics, hydrogen bonds, hydrophobic interactions, ionic interactions

## Abstract

This study aims to investigate the interactions appearing when the beta-2-glycoprotein-1 binds to a lipid bilayer. The inter- and intra-molecular forces acting between the two macromolecular systems have been investigated using a molecular dynamics simulation method. The importance of water bridges has also been addressed. Additionally, the viscoelastic response of the bilayer has been studied. In detail, the (saturated-chain) 1,2-dipalmitoyl-sn-glycero-3-phosphocholine (DPPC) and (unsaturated-chain) 1-palmitoyl-2-oleoyl-sn-glycero-3-phosphoethanolamine (POPE) bilayers have been chosen to test their behavior near the protein. Both of the lipids have a polar head but different chemical structures and are similar to the main phospholipids present in the synovial fluid. This study is meaningful for further explaining the worsening friction properties in articular cartilage, as the inactivation of phospholipid bilayers by beta-2-glycoprotein-1 is believed to be a cause of the destruction of cartilage in most rheumatic diseases and osteoarthritis. It was found that the protein binds stronger to the DPPC bilayer than to the POPE, but in both cases, it has the potential to change the local bilayer stability. Nevertheless, the binding forces are placed within a small area (only a few lipids contribute to the binding, creating many interactions). However, together, they are not stronger than the covalent bonds between C–O, thus, potentially, it is possible to push the lipids into the bilayer but detaching the lipids’ heads from the tail is not possible. Additionally, the protein causes water displacement from the vicinity of the bilayer, and this may be a contributor to the instability of the bilayer (disrupting the water bridges needed for the stabilization of the bilayer, especially in the case of DPPC where the heads are not so well stabilized by H–bonds as they are in POPE). Moreover, it was found that the diffusivity of lipids in the DPPC bilayer bound to the protein is significantly different from the diffusivity of the ones which are not in contact with the protein. The POPE bilayer is stiffer due to intramolecular interactions, which are stronger than in the DPPC; thus, the viscous to elastic effects in the POPE case are more significant than in the case of the DPPC. It is, therefore, harder to destabilize the POPE bilayer than the DPPC one.

## 1. Introduction

Every living cell consists of lipids and proteins. Proteins transiently attached or directly embedded in the plasma membrane perform many important cellular functions, such as transport of organelles or molecules, membrane reorganization, and signaling. The meaning of this class of proteins can be inferred based on their relative importance, as they represent 40% of all cellular proteins [1,2]. Modulation of membrane surface protein activity often occurs after conformational changes resulting from binding to the surface of the cell membrane [3]. Interactions with specific lipid classes have also been reported to directly modulate the activity of transmembrane proteins, such as enzymes involved in lipid metabolism or ion channels [4]. Understanding the mechanisms of selected proteins, such as beta-2-glycoprotein-1 (β2-GP-1), which recognize and bind to their lipid partners, is the real challenge. It would provide insights into the functionality of these proteins and may facilitate the future development of drugs to treat various pathological conditions.

**Figure 1:**
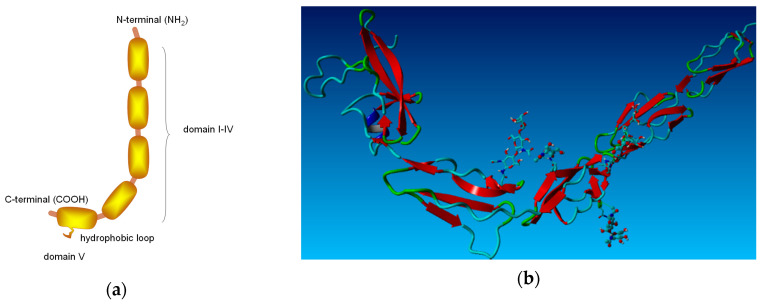
(**a**) Crystal and **(b**) initial structure of beta-2-glycoprotein-1 (β2-GP-1) (ribbon representation).

β2-GP-1 is a soluble 50–54 kDa glycoprotein. The structure of β2-GP-1 is shown in Figure 1. This protein takes the form of a crystal structure that mimics a fishhook-like appearance with a dimension of 130 Å in the vertical direction and 85 Å in the horizontal direction. It is present in human blood plasma at a concentration of 150–300 μg/mL. The isoelectric point of β2-GP-1 is at pH in the range 5–7, so the protein is negatively charged at physiological pH = 7.4. Each protein molecule has five domains: four structurally similar domains and a fifth domain, which contains an extra c-terminal loop, which is necessary for the phospholipid binding. The β2-GP-1 protein can interact with phospholipid membranes by electrostatic interactions and hydrophobic loops. The ionic and hydrophobic interaction of the fifth (and sometimes fourth) domain is important for binding β2-GP-1 with the phospholipid membranes [5,6,7].

From a technical point of view, a conformational change within β2-GP-1 can exist in the closed circular conformation of plasma β2-GP-1 and the open fishhook-like structure. In the second case, β2-GP-1 is complexed with an antibody directed against domain (I). The phospholipid-binding site is mainly domain (V) of β2-GP-1 (Figure 1a), and it contains some positively charged amino acids. In plasma, the protein is present as a closed circular protein in which domain (I) interacts with domain (V) [8,9]. Following interactions with anionic surfaces, the protein opens up and adopts the fishhook-like conformation, resembling a crystal structure [10]. When β2-GP-1 encounters cells that (temporarily) expose anionic phospholipids on their surface, β2-GP-1 binds to these phospholipids (-PO_4_^−^). This phenomena results in a conformational change of the protein. Such a change presents an antigenic determinant of the epitope for the autoantibodies, which is generally shielded from recognition [10]. The autoantibodies bind and stabilize β2-GP-1 in its fishhook-like conformation. Although it is not fully understood, the function of β2-GP-1 seems to be related to blood coagulation and the removal of phosphatidylserine- and cardiolipin-containing liposomes from circulation [11,12,13]. Many diseases appear when this protein folds into this specific antibody conformation, e.g., modulation of platelet thrombosis, atherosclerosis, and autoimmune diseases, e.g., antiphospholipid syndrome [14,15,16].

β2-GP-1 has also been found in synovial fluid, and it is believed to impact the mechanism of lubrication seen within joints [16,17]. Synovial fluid is indeed a very complex system. It consists mainly of hyaluronic acid, phospholipids, and lubricin, but also it contains various proteins [18,19]. The interactions of the components have a complicated multiscale and synergistic nature. Such interaction forms a lubrication mechanism that is still not well described [20].

For this reason, it is important to analyze the dynamics of each component and the synergy between them [21]. β2-GP-1, in its specific fishhook-like conformation, binds to the phospholipid bilayer, which covers the articular cartilage [17]. While many studies describe that such binding takes place, there is still a lack of information on detailed analyses of the interactions appearing between the protein and the bilayer and the number of such events.

In many studies, the interaction of β2-GP-1 (and also different charged proteins) with liposome membranes containing negatively charged phospholipids (cardiolipin, phosphatidylserine, phosphatidylinositol) has been discussed [22,23,24,25]. However, protein interaction with the zwitterionic lipids (phosphatidylcholine, phosphatidylethanolamine, and sphingomyelin) has rarely been studied [9,23,26,27]. Such interactions can be explained in terms of electrostatic forces between the charged surface and charged proteins. Further studies of lipid monolayers in this protein’s proximity have shown that β2-GP-1 also interacts with neutral dipalmitoylphosphatidylcholine. These interactions showed an increase in the surface pressure of the lipid monolayer [27]. In Reference [7], the authors described the interactions by observing an increase in the modulus of elasticity, which slightly depends on the concentration of β2-GP-1.

Considering the importance of β2-GP-1 adsorption at the neutral or non-neutral lipid bilayer, significant effort has been put into understanding their structure and dynamic behavior. For practical reasons, two different types of lipids were chosen to analyze their interactions with β2-GP-1: 1,2-dipalmitoyl-sn-glycero-3-phosphocholine (DPPC) and 1-palmitoyl-2-oleoyl-sn-glycero-3-phosphoethanolamine (POPE). The argument for such a choice is the fact that the surface layer of the articular cartilage contains 41% phosphatidylcholine, 27% phosphatidylethanolamine, and 32% sphingomyelin [28]. Therefore, we chose to study phosphatidylcholine and phosphoethanolamine as components similar to those in the synovial fluid. The separate studies on POPE and DPPC bilayers (not mixed like in the biological membrane) were crucial to gain baseline help to analyze differences between the bindings’ interaction maps for different kinds of the bilayers that differ in head-groups and tail-groups. The analysis of the membrane in which composition is closer to the real one is one of the goals of our further work.

This study aims to investigate the interactions between β2-GP-1 and DPPC or POPE using molecular dynamics (MD). The computer simulations were performed in the presence of explicit water with monovalent ions (Na^+^, K^+^, Cl^−^) to check the influence of the solvent on the studied interactions in physiological conditions.

Such investigation follows the mainstream approach as MD simulation’s contribution to molecular biology, and drug discovery has sharply expanded in recent years. These simulations capture the expression of proteins and other biomolecules in full atomic detail and with an excellent temporal resolution. Simulations have proved to be a valuable contribution to deciphering the functional mechanisms of proteins and other biomolecules. They also helped discover the structural basis of many diseases and allow for the design and optimization of small molecules, peptides, and proteins [29]. In this work, we used full-atom MD, looking deeply into the interaction map between the protein and bilayer and inside the intramolecular interactions inside β2-GP-1 and inside the bilayers. We also present the investigation results toward the bilayers’ viscoelastic properties, taking into account the differences between the layer connected to the protein and the layer without contact with the protein.

The paper is organized as follows. In Section 2, we describe the molecular dynamics simulation and explain the calculation of all of the parameters used to explore the characteristics of the binding of β2-GP-1 to the DPPC and POPE bilayers. In Section 3, we present the results and their discussion regarding whether they can enrich the understanding of the protein’s influence on the bilayer’s stability. The paper closes with the conclusions in Section 4.

## 2. Materials and Methods

This study evaluated the interactions between the molecules in a model system consisting of a lipid bilayer and protein. Based on the results of all-atom molecular dynamics simulations, the research was performed using the AMBER03 force field [30] to evaluate the interactions between the molecules in the model system. The YASARA Structure software (Vienna, Austria) was chosen to perform all simulations. The structure of β2-GP-1 was taken from PubChem (Protein Data Bank (PDB) ID: 1C1Z–5280 atoms) [31]. The lipids used for this study were DPPC (130 atoms) and POPE (125 atoms). In both cases, the lipid bilayer consists of 288 lipids (144 lipids in each layer). Both bilayers’ structures came from the studies presented by Bioinformatics Group of the International Scientific and Educational Center of National Academy of Sciences of the Republic of Armenia in Reference [32]. The DPPC and POPE bilayers containing 72 lipids were copied four times to obtain a structure to build a contact area between the protein and the bilayer freely. The bilayer was simulated for 5 ns to allow the four bilayer parts to assemble into one continuous structure of thickness of about 60 Å for DPPC (with water penetration zone of thickness about 20 Å), and about 55 Å for POPE (with similar water penetration zone). After this time, the protein molecule was placed next to the bilayer without influencing its structure. The protein structure was minimized before the MD simulations.

Periodic boundary conditions were applied to mimic the infinity of the system and ensure that the edges do not influence the system’s stability. For the isobaric-isothermal ensemble, all-atom simulations were performed under the same conditions: temperature 310 K and pH = 7.0. Two water-based solutions were simulated: 2% NaCl aqueous solution and 2% KCl (TIP3P water model was used [33]), with a time step of 2 fs. The simulations lasted for 50 ns. The obtained simulation results contained one step for every 25,000 steps, providing 1000 samples overall. The results are presented separately for the two different ion additions (Na+ and K+). Such an approach was applied to check if there are any differences in the simulation results. The Berendsen barostat and thermostat with a relaxation time of 1 fs were used to maintain constant pressure and temperature [34]. The β2-GP-1 was placed near the bilayer in a YASARA simulation box of size: X: 95 Å, Y: 86 Å, Z: 196 Å, and then the system was equilibrated for 10 ns. The simulations were run for 50 ns and repeated 5 times for each condition. The simulation runs have had similar initial conditions that have differed slightly in simulation temperature (at third decimal digit). Simulation temperature usually serves for the YASARA software as the random number seed. Next, the results were averaged to show results reliable from a statistical point of view. We started the simulations from the situation where the protein was already positioned close to the bilayer (soft bind), and next, we checked how it behaved over time (how this binding relaxed).

**Figure 2:**
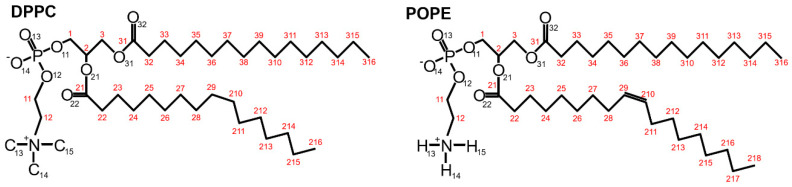
Chemical structure of DPPC and POPE molecules with oxygen and nitrogen atoms (to underline the difference between the heads of both lipids, the proper carbon atoms (C13–C15) and chosen hydrogen atoms have been included). Names of atoms are taken from the PDB. Red numbers are numbers of carbon atoms [35]. The structure can be imagined as a hydrophilic “head” with two hydrophobic “tails”.

The presented results aimed to improve the binding process’ understanding between a lipid bilayer and β2-GP-1 [16]. We computed hydrophobic-polar (HP) interactions, ionic interactions, and hydrogen bonds (H–bonds) inside the system to analyze the stability and dynamics of the system’s structures. We considered intra- and inter-molecular bonds separately. The intramolecular interactions are the sum of the interaction between all atoms inside the β2-GP-1 and DPPC/POPE, whereas the intermolecular interactions are the interactions between the two molecules: the phospholipids and the protein. The chemical structures of DPPC and POPE are shown in Figure 2.

According to the YASARA definition, the hydrogen bond is formed when the hydrogen bond energy is greater than 6.25 kJ/mol, 25% of the optimum value of 25 kJ/mol. The exact formula has been described in the YASARA manual and previously in References [20,36,37,38]. Additionally, we identified water bridges created by water bonding between two different molecules because inside the POPE bilayer, there are no H–bonds, and only water mediates between interactions.

The parameters that can tell us more about the bilayer stability, which we have chosen to present, are the mean squared displacement (MSD) and the macromolecules’ entropy.

The MSD of a bilayer’s molecule at time step *t* is obtained from the lipids’ trajectories in the standard way:

(1) $$MSD\left(t\right)=\frac{1}{N}\overset{N}{\underset{i=0}{\sum }}{\left|{\vec{r}}_{i}\left(t\right)-{\vec{r}}_{i}\left(0\right)\right|}^{2},$$

where *N* is the number of lipids in the simulation box and ${\vec{r}}_{i}$ is the position vector of the geometrical center of a molecule *i*. To observe a difference between the behavior of the lipids’ layers (the one in contact with the protein and the one without the contact), the MSD was computed separately for the upper and lower lipids’ layers. The time evolution of the MSD of the lipid particles (cf., Equation (1)) characterizes the viscoelastic properties of the bilayer [39]. It is related to the environment’s mechanical response functions through the generalized Stokes-Einstein relation [40], and in three-dimensional (3D) Euclidean space, it follows the relationship [41,42]:

(2) $$MSD\left(t\right)=6D_{\alpha }t^{\alpha },$$

where *D_α_* is a generalized self-diffusion coefficient (a constant that does not depend on time and is of the dimension of [*D_α_*] = cm^2^/s^α^) [43,44], exponent α = 1 represents normal diffusion, and α ≠ 1 represents anomalous diffusion (when α > 1, a super-diffusion process appears, and when α < 1, we have sub-diffusion).

From the time-dependent MSD, the linear viscoelastic complex shear moduli, G^*^, of a complex system can be obtained [45]. Each lipid molecule was approximated as a spherical object of radius *R_g_*, where *R_g_* is the mean radius of gyration computed overall for lipid molecules, as described in Reference [46]. The G^*^ moduli can be obtained using the generalized Stokes-Einstein relation in the Fourier domain:

(3) $$G^{*}\left(\omega \right)=\frac{k_{B}T}{\pi R_{g}i\omega F\left\{MSD\left(t\right)\right\}},$$

where *k_B_* is the Boltzmann constant, *T* is temperature, and ω is frequency. It can be estimated as [44]:

(4) $$G^{*}\left(\omega \right)\approx \frac{k_{B}T}{\pi R_{g}MSD\left(\frac{1}{\omega }\right)\Gamma \left[1+\alpha \left(\omega \right)\right]},$$

where $\alpha \left(\omega \right)={\frac{dln\langle MSD\left(t\right)\rangle }{dlnt}|}_{t=1/\omega }$ and the real and imaginary parts of the complex viscoelastic modulus can be obtained from the relations: $G^{\prime }\left(\omega \right)=G^{*}\left(\omega \right)cos\left(\frac{\pi \alpha \left(\omega \right)}{2}\right)$ and $G^{\prime \prime }\left(\omega \right)=G^{*}\left(\omega \right)sin\left(\frac{\pi \alpha \left(\omega \right)}{2}\right)$, where G^*^(ω) = *G*’(ω) + i*G*’’(ω). G^*^(ω) determines the stress induced in a material upon application of an oscillatory shear strain at frequency ω. The real part of the shear modulus, *G*’(ω), is the storage modulus, and is the elastic susceptibility–the elastic component of the stress. The imaginary part of the shear modulus, *G*″(ω), is the loss modulus, and is the viscous component of the stress.

In this research, Schlitter entropy was used [47]. It was calculated from the mass-weighted covariances of a single lipid inside the bilayers and the averages over all lipids. In this approach, the entropy of the conformational ensemble of the single lipid molecule can be computed by the following formula:

(5) $$S=k_{B}\cdot \ln\;\det\left(1+\frac{k_{B}\cdot T\cdot e^{2}}{\hbar^{2}}\cdot M⊙\sigma \right),$$

where **1** is the unity matrix, ***M*** is the mass matrix (containing atomic masses in the diagonal), *e* is Euler’s number, and ***σ*** is the symmetrical covariance matrix whose elements are given by:

(6) $$\sigma_{ij}=\langle \left(x_{i}-\langle x_{i}\rangle \right)\cdot \left(x_{j}-\langle x_{j}\rangle \right)\rangle .$$

The mean value in angular brackets is an average of all positions of atoms *i* and *j* in Cartesian space (separately for the x, y, and z coordinates) calculated for a given time. The assumption needed for this computation is that fluctuations are normally distributed around the mean, allowing the covariances to be estimated from a multidimensional Gaussian distribution [48]. Otherwise, the covariance matrix does not yield all information tied to the cross-correlation of the data, and other measures such as higher-order moments or cumulants can be beneficial in a more detailed research. For justification, see the Introduction in Reference [49]. The covariance matrix was computed for all atoms in the single lipid molecule; hence, its size is 130 × 130. Realizations are tied to the number of simulation steps—for each 1 ns of the simulation time, we have 20 steps. Furthermore, the x, y, and z coordinates of the atom positions are taken separately for the mean subtraction procedure. Next, these zero mean series are concatenated for the computation of the covariance matrix, e.g., for 1000 steps, we have 3000 realizations (1000 × 3 directions). Observe that for a small number of steps, we have a small number of realizations, and the covariance matrix is singular. Here, the identity matrix addition in Equation (3) makes the determinant non-zero. We should also mention, from the technical point of view, that the simulation software sometimes produces artefact jumps of the size of the simulation box. These jumps were detected automatically (by investigating the deviation from the first step) and leveled. Although, as discussed in Reference [47], the spatial covariance may overestimate the entropy (in the case of molecules which are not frozen and making translational motions), we are interested in the shape of the entropy vs. the *t* curve rather than its particular value.

## 3. Results and Discussion

**Figure 3:**
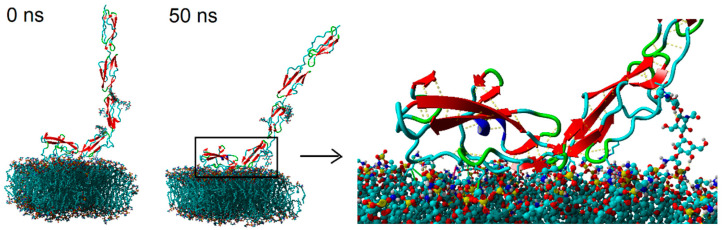
Initial and final structures of POPE bilayer and β2-GP-1 at the start of the simulation (0 ns) and after 50 ns, for 2% NaCl solution of water. Light blue atoms represent carbon, dark blue—nitrogen, red—oxygen, yellow—phosphorus, and white—hydrogen (solvent molecules, and hydrogen atoms in lipids are hidden). Note that the periodic boundary conditions, present during simulations on each wall, are switched off to show the molecules as non-fragmented (i.e., the simulation space was expanded for presentation purposes). Yellow dotted lines show the hydrogen bonds in the protein.

The exemplary initial and final structures of the system are presented in Figure 3. Due to the preliminary observation that no significant changes occurred in the system during the simulation time, we looked more deeply into the processes occurring both in the contact zone between the β2-GP-1 and the bilayer, and inside the bilayer. The simulations continued until the system’s energy stabilized—in all cases, the stabilization of the system energy could be seen at the simulation time of around 40–50 ns (cf. Supplementary Materials Figure S1). To test if that was a good time to stop the MD simulations, we computed the bilayers’ conformational entropy.

**Figure 4:**
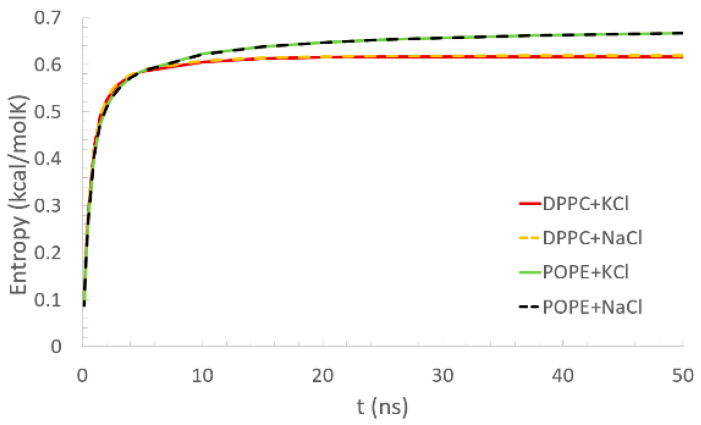
Mean conformational entropy of a single lipid molecule as a function of time (coming from the bilayer in the vicinity of the protein). Lines which show entropy for the same bilayer but with a different addition of ions in the solvent overlap.

As a function of time, the conformational entropy of the bilayers was calculated according to Equation (3) and is presented in Figure 4. All given curves reach a plateau: in the case of the DPPC, after 10 ns, and for the POPE, after about 20 ns. In the case of the DPPC, the plateau equaled about 0.62 kcal/molK. For the POPE, the entropy continued to slightly increase for the whole simulation time. The rate of this increase, however, decreased with the simulation time. At the maximal simulation time, the entropy equaled about 0.66 kcal/molK. Based on this observation, we can say that longer simulations are needed to equilibrate the POPE than DPPC bilayers, but in both cases, 50 ns was enough to observe the interactions’ formation. Similar results were obtained for the DPPC bilayer by Wustner et al. [48], where the results come from Monte Carlo simulations of the bilayer self-organization. They present there the methods as a good indicator of equilibration of the system. Such a short simulation time is enough as we are not focusing on observing the conformational changes of the protein. The involvement of monovalent Na+ or K+ ions in the solvent does not influence the lipid bilayers’ conformational entropy.

As is written in Section 2, the covariances matrix is needed to compute the entropy (cf., Figure 4, and Equations (3) and (4)). Such an approach assumes that fluctuations are normally distributed around the mean, allowing covariances to be estimated from a multidimensional Gaussian distribution. The careful analyses of histograms (see Supplementary Materials Figure S2) demonstrate that the bivariate distribution of an example pair of *i* and *j* atoms are not strictly Gaussian. However, they look similar to Gaussian, and for most cases, there is no strong evidence of tail correlations (or tail dependencies). Hence, we used the covariance approach as the approximation revealing the state-of-the-art line. Some evidence of multimodality in univariate histograms may be caused by the fact that the x, y, and z coordinates’ data are taken separately.

Furthermore, analyzing the histograms mentioned above in more detail shows that the head-atoms’ coordinates are much more correlated. Hence, head-atoms seem to move together in a relatively rigid manner. The tail-atoms’ coordinates are much less correlated. Thus, the tails are more flexible, and the tail-atoms’ movements are less synchronous. Such rigidity of movement of most of the head-atoms confirms the existence of forces of a hydrophilic nature which stabilize the lipid membrane. In Reference [50], it is explained that the main mechanism of such stabilization is a result of the creation of strong water bridges built between hydrophilic molecules. How much the bilayer is hydrated is shown by the number of H–bonds between water and the bilayer (per one lipid molecule) in Reference [51]. The simulations revealed about five H–bonds with water per one lipid molecule for the DPPC and more than six for the POPE. If the water structure is changed due to the presence of, e.g., protein, the local stabilization of the head-atoms can be disturbed because different interactions replace the water bridges. That is why it is important to indicate how many such events can appear and how many lipids could be affected. In Reference [52], the authors analyzed intermolecular interactions inside two types of bilayers. We prepared similar investigations, but for a system extended by β2-GP-1.

**Figure 5:**
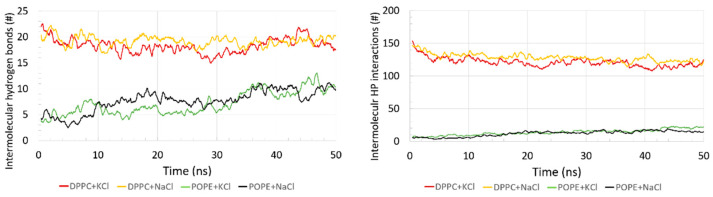
Time evolution of the number of intermolecular H–bonds (left) and intermolecular HP contacts (right) between protein and bilayers.

In Figure 5, a number of intermolecular H–bonds and HP interactions are shown. The number of intermolecular H–bonds between the protein and bilayers was small relative to the intramolecular ones (see, Supplementary Materials Figure S3). For the DPPC, the number of these bonds was greater than for POPE and oscillated in the range 15–23. The number of intermolecular H–bonds for the POPE increased from 4 at the beginning of the simulation time to 10 at the end. The number of lipids involved in the intermolecular H–bond binding increased from 11 to 13 for the DPPC (each lipid created 1 to 4 H–bonds with the protein) during the simulation time and from 4 to 8 for the POPE (each lipid created 1 to 3 H–bonds with the protein). Recall that the number of all lipids in the layer on the side bound to the protein is 144; thus, only a small percentage participated in the binding between the protein and the bilayer. We also observed that the DPPC did not form any intramolecular H–bonds between lipids (see, Supplementary Materials Figure S3). Conversely, the POPE formed a lot of these bonds (between nitrogen atoms and oxygen atoms), and the number increased from 205 at the beginning of the simulation to 240 at the end of the simulation. This fit the exponential slope y(*t*)~*t*0.04.

**Figure 6:**
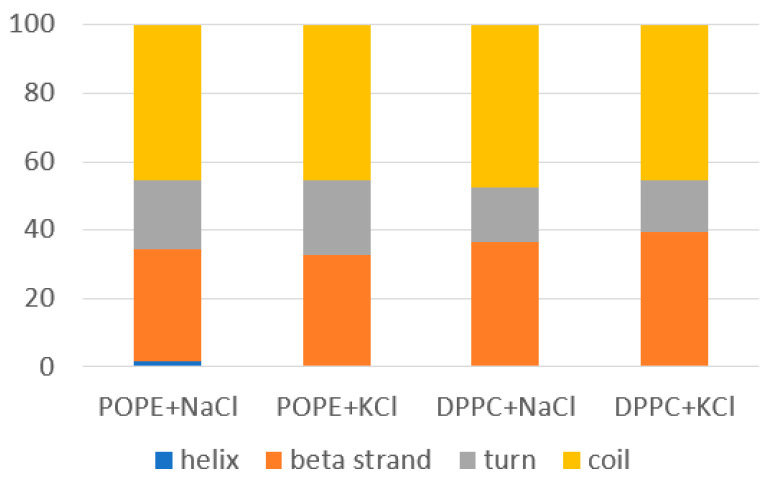
Secondary structure of the β2-GP-1 bound to the bilayer after 50 ns of simulations.

The protein contained a greater number of intramolecular H–bonds when binding to the POPE bilayer than to the DPPC (see, Supplementary Materials Figure S3 (right)). The difference was around 10 interactions. This fact could result from the compensation of the number of intermolecular H–bonds between the protein and bilayers. The difference was similar both for DPPC and POPE. Looking more deeply into the protein changes, placed 50 ns in the bilayer’s vicinity, we analyzed the percentage content of the secondary structure in the protein (see Figure 6). Time evolution of the secondary structure in each case is characterized by slight oscillations only (see Supplementary Materials Figure S4).

Further, the small but visible difference can be seen when looking on the number of turns and beta strands in the protein bound to the POPE compared with the protein bound to DPPC. For DPPC, there was a slightly lower percentage of turns in favor of the coils and beta strands. For POPE, from time to time, 1.2–1.8% helixes appear. Note that in YASARA, a ‘turn’ is a stretch of four residues that are not part of helices or strands and form a hydrogen bond between the O of the first and the last residue’s NH. The results show that the protein’s V domain slightly reorganizes due to interactions with the bilayer. The protein’s radius of gyration also oscillated in time around the value of 48 Å, but the oscillations sometimes jump even to around 40 Å.

**Figure 7:**
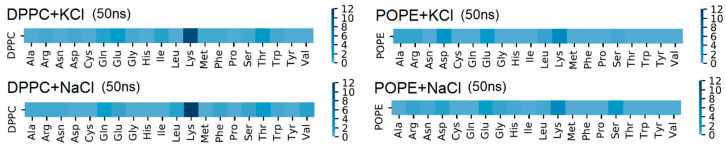
Map of β2-GP-1 amino acid H–bonding phospholipid molecules (number of the bonds) after 50 ns of simulation time: left for DPPC, right for POPE.

The amino acid responsible for the greatest number of intermolecular H–bonds was Lys (see Figure 7), which agrees with experimental results [9]. The first amino acid (in the protein sequence) which binds the protein by H–bond to bilayer was 246 Lys (V domain) for DPPC and 193 Asp (IV domain) for POPE. The last one for DPPC is 318 Thr and 312 Ser-314 Ala for POPE. The detailed maps of the interactions found are presented in Figure 7 and Supplementary Materials Figures S5 and S6. Looking from the bilayer side, most of the H–bonds were created by O13 and O14 (so, phosphide oxygens placed in the head-group of the lipid, cf. Figure 2). In the case of DPPC, all of the lipids’ atoms that created H–bonds with the protein were oxygens, thus acceptors. In POPE, most of the lipids’ atoms that created H–bonds with the protein were mostly oxygens (acceptors), but nitrogen atoms donors created H–bonds. The number of donors was approximately two times lower than acceptors.

Other important interactions for the self-assembled structure formation were the hydrophobic-polar (HP) interactions. They are solvent-mediated forces, and they are crucial for stabilizing molecular structures. The number of intermolecular HP interactions as a function of time are presented in Figure 5 (right).

Similarly, as with the H–bonds, the number of intermolecular HP interactions between the protein and bilayer was greater for the DPPC than for the POPE. The DPPC slightly decreased in time, while for the POPE, it slightly increased in time. The number of lipids involved in the intermolecular HP binding decreased from 28 to 22 for the DPPC (each lipid created 1 to 35 HP interactions with the protein) and increased from 2 to 7 for the POPE (each lipid created 1 to 9 HP interactions with the protein). Thus, the protein bond is stronger to the DPPC by HP interactions, similar to the case of H–bonds, which results from the differences in the structure of the POPE and DPPC heads. In the case of POPE, there is no difference in the number of intermolecular HP interactions between the systems with different ions.

On the contrary, for DPPC with NaCl, we can see a slightly greater number of the intermolecular HP interactions, but this observation needs further research to present the reason for such discrepancy. It could affect some of the ion’s interaction with the protein and the bilayer via ionic interactions (see, Figures S7 and S8 in Supplementary Materials). Because of phospholipids’ construction, which consists of hydrophobic alkyl tails and hydrophilic phosphate heads, phospholipids could easily form bilayers through hydrophobic interactions in water [53].

**Figure 8:**
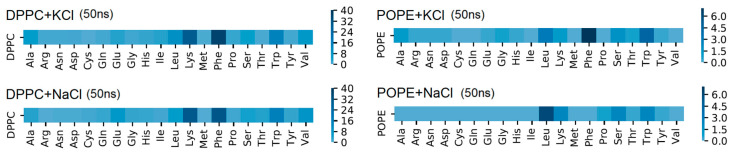
Map of HP interactions between β2-GP-1 amino acids and phospholipid molecules after 50 ns of simulation time: left for DPPC, right for POPE. Pay attention to the color scale, which varies between POPE and DPPC for better visibility.

The number of intramolecular HP interactions inside bilayers was of the order of 10^4^, which is much larger than all of the other interactions in the system (see Supplementary Materials Figure S9). In the case of the DPPC, there was a big, exponential increment in the number of intramolecular interactions (with slope y(*t*)~*t*^0.05^). In the POPE case, the number of intramolecular HP interactions increased linearly in time with a gentle slope. The difference between these two curves’ behavior comes from the difference in the chemical structure of the bilayers. POPE has a double bond between atom C29 and C210, and HP interactions prevailed between the ends of the lipid tails (see Supplementary Material Figure S8 (right) and the dark space between C218 and C316). The rest of the molecule interacted with others via water bridges. In the case of DPPC, there is no double-bond, and there are fewer water bridges. The tails built HP interactions between each other (sn-1 and sn-2 chains; see Supplementary Material Figure S8 (right) and the dark space between carbon atoms C22-C26 and C32-C35). There was also a greater number of intramolecular HP interactions inside the protein bound with the POPE than with the DPPC. The difference was stable across the whole simulation period—equal to about 40 HP interactions (see, Supplementary Materials Figure S10). Maps of the HP interactions between the protein and bilayers are shown in Figure 8 and Supplementary Material Figure S11. We can see the discrepancies between those two bilayers in Figure 8. There are also some slight discrepancies between the results where simulations were performed with different salt addition in the solvent, but as was mentioned above, this observation is not crucial and needs further research. Different parts of protein created HP interactions with the bilayer. For the DPPC, most interactions came from Lys (charged), Phe (nonpolar), and Trp (nonpolar). For the POPE, most interactions came from Leu, Phe, and Trp. The first amino acid (in the protein sequence) which binds the protein by HP interactions to DPPC and POPE bilayers was 219 Tyr (IV domain). It is important to observe that more stable groups of bonds start from amino acid number 244 to 270 (Ser to Gly; domain V) and 300 to 318 (Thr to Thr) in the case of DPPC and from 311 to 315 (Ser to Phe) for POPE (more detailed information can be found in the Supplementary Materials in Figure S11). The most dominant, in terms of HP interactions from the side of the bilayer, were carbon atoms, of which the most important were C11 and C12 (see, Supplementary Materials Figure S9).

**Figure 9:**
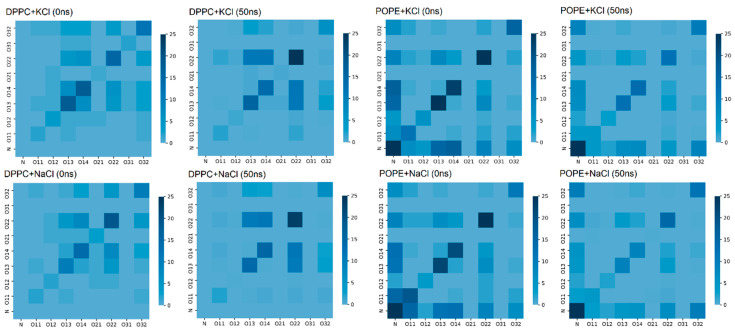
Map of water bridges between bilayer atoms: before (0 ns) simulation and after (50 ns) molecular dynamics (MD) simulation.

Water bridges between lipids in the bilayer are very important for the stability of the bilayer. The atoms forming such bridges are presented in Figure 9 in the form of a map: both at the beginning of the simulation and after 50 ns of simulation. In the case of the DPPC, we can see that the number of bridges increased during the simulation and occurred mostly between oxygens O22-O22 (cf., Figure 2).

In the POPE case, the number of water bridges decreased during 50 ns of simulation, but it was still greater than in the case of the DPPC. In Figure 9, for POPE, one can see that our self-assembling system favored bridges between the N–N and N–O atoms. The bridges between O22-O22, O13-O13, and O14-O14 were also present, but some were destroyed during the simulation—a different type of interaction probably replaced them. Nevertheless, in both cases, the bridges appeared in the lipids’ hydrophilic heads, stabilizing them. In Reference [50], the author stated that hydrophilic forces play a crucial role in protein folding and assure protein stability, not the hydrophobic ones. He proved that two hydrophilic parts of a protein, when appropriately located, build a strong interaction by creating an H–bond-bridge between them. In our simulations, we can observe similar bridges in the hydrophilic part of the bilayer.

**Figure 10:**
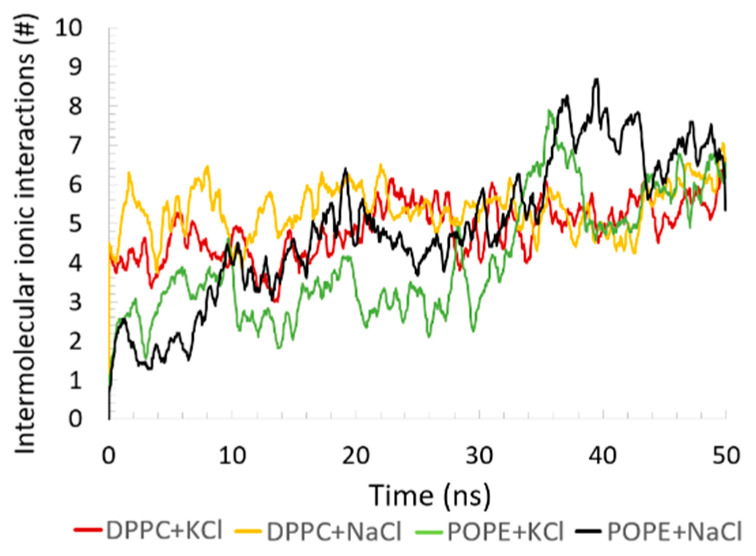
Intermolecular ionic interactions between the protein and the bilayers as a function of time.

Although ionic interactions were present in the system, the number of such interactions was small compared to the H–bonds and HP contacts. Between the protein and bilayer, there are a few such interactions (see Figure 10), and the type of bilayer did not change this picture much. Most of the interactions were inside the POPE bilayer—they also stabilized the bilayer. Moreover, we checked how ions added to the water-based solvent influenced the simulation results. All simulation results presented so far were independent of (or slightly dependent on) which cation was added—Na^+^ or K^+^. We checked the fraction of the ions from the solvent that created ionic interactions with the simulated molecules. The number of ionic interactions coming from the added ions did not change in time—it only oscillated. The number of added atoms in each simulation were: DPPC+NaCl — 250Na^+^, 255Cl^−^; POPE+NaCl — 278Na^+^, 284Cl^−^; DPPC+KCl — 197K^+^, 202Cl^−^; POPE+KCl — 219K^+^, 225Cl^−^. Note that the number of anions and cations is similar but not the same in each model system. At the stage of solvent preparation, ions are placed at the locations of the lowest/highest electrostatic potential until the simulation cell is neutralized and the requested ion mass fraction is reached. For the DPPC, only 2.0% and 2.5% reacted with the protein-bilayer system for the POPE. For the DPPC, the Na^+^ and K^+^ most often attached P atoms (approximately 8 interactions in the case of Na^+^ and 5 in the case of K^+^) but also Na–C and N–Cl ionic interactions can be found (1 or 2 such interactions). For the POPE, the Cl^−^ anion ionic interactions with N prevailed (11 interactions in the case of Na^+^ and 7 interactions in the case of K^+^), and P-Na^+^ (or P-K^+^) interactions were present (1 to 4 such interactions).

Partially summarizing the results presented above, from the detailed analysis of the number of intermolecular bonds and assuming they are dynamic, one can conclude that the protein creates more connections to DPPC than POPE. In both cases, only a few lipids participated in the binding, but they created many interactions; thus, the binding energy was quite strong. The intramolecular interactions analysis provided information about the changes inside the bilayer and protein systems. Thus, there were no significant changes in the protein conformation within the studied 50 ns of the computer simulations (a stable number of intramolecular interactions inside the protein was noticed), and the percentage content of the protein’s secondary structures only displays some oscillations in time. Nevertheless, it was not true for the bilayer, where the number of intramolecular interactions of various types grew significantly. This phenomenon is usually connected with increased stabilization of the bilayer [52] (especially in POPE). The effect of building intramolecular connections inside the bilayer and the presence of water bridges at the surface of the bilayer competes with the effect of destabilizing the bilayer by protein presence. The information on how deep the protein penetrates the bilayer can help understand the system’s behavior. In the case of the DPPC, the protein penetrates the bilayer to the depth of 27–30 Å; thus, it penetrates about half the bilayer’s depth (see, Supplementary Materials Figure S12). The amino acids which were found the deepest were 313 Leu and 315 Phe. In POPE, the protein penetrates only the shallow external layer—2.5–3.5 Å depth—and the deepest dived amino acids were 313 Leu, and 251 or 305 Lys (depending on the simulation repetition). To obtain information about the penetration depth and membrane thickness, the electron density profiles have been used (see, Supplementary Materials Figure S13). It is shown that at the beginning of the simulations, water pockets inside the bilayer were found. Thus, the initial bilayer thicknesses for DPPC and POPE were greater than the ones obtained after 50 ns. This results from an even distribution of water molecules over the whole available space at the start of MD simulation. After reaching the system’s equilibrium in the preparatory stage of the simulation, they were still present. However, the water was pushed out during the first few nanoseconds of the simulation process, and they became almost absent inside the bilayer after about 25 ns for DPPC and 15 ns for POPE. Thus, during simulation time, the water was pushed away from the bilayer and the bilayer shrunk by about 5 Å. A detailed analysis of the two bilayer systems and water influence on their creation has been presented in Reference [51]. Such a density profile can be obtained experimentally by the combined use of X-ray and neutron diffraction measurements [54] and provides comparable results.

Going back to the conformational entropy, we can try to explain the dynamic of the entropy in relation with the number of inter- and intra-molecular interactions between the bilayer and protein. In Figure 4, it can be seen that the entropy parameter for DPPC bilayer stabilized faster. It could be a result of much higher number of intermolecular interactions of DPPC lipids with the protein than in POPE. It is despite an important influence of hydrophobic heads in which POPE is better stabilized by intramolecular H–bonds (recall that no such interactions were present in DPPC bilayer). If the tail-effect prevails head-effect, the POPE membranes would stabilize faster than DPPC, but having the opposite effect, it can be seen that head-effect prevails.

**Figure 11:**
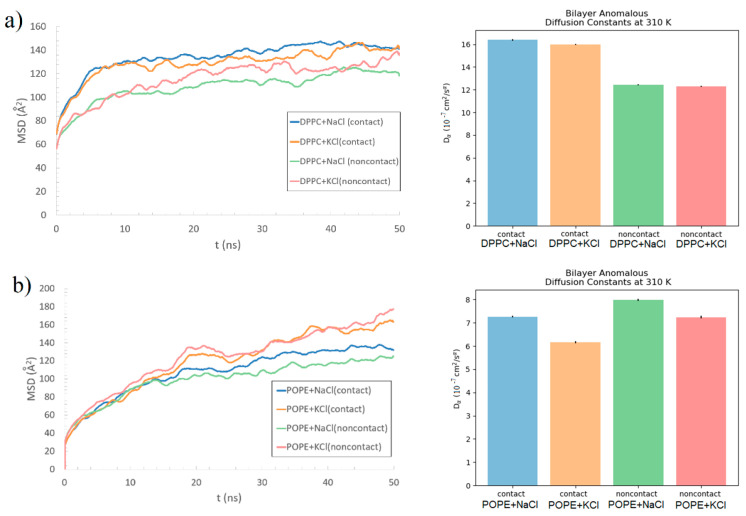
Evolution of the MSD of the lipids in time (left) and fitting values of diffusion coefficient *D_α_* (right) for two lipid bilayers: (**a**) DPPC and (**b**) POPE, averaged over all lipid molecules for all iterations of the simulation. Black lines on the bar graph show the standard deviation (STD) of the fitting parameters. All results were obtained for the bilayers with the presence of the protein bound to it.

**Figure 12:**
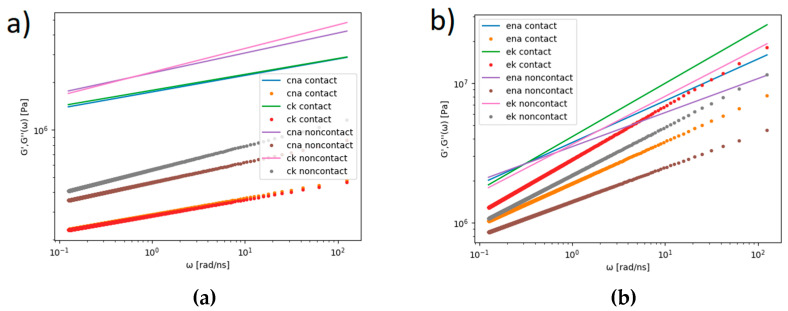
Complex viscoelastic moduli, G’—dot symbols and G’’—cross symbols, for DPPC (**a**) and POPE (**b**). Abbreviations: ck—DPPC + KCl, cna—DPPC + NaCl, ek—POPE + KCl, ena—POPE + NaCl.

To check the bilayer stability, e.g., if the protein pulls the lipids, the lipids’ MSD in the bilayer was computed. As macromolecular systems often exhibit anomalous behavior, we tested the scaling relation *MSD(t)~t^α^* (cf., Equation (2)), where *α* is the scaling exponent fitted from the data. The MSD, as a function of time, is shown on the left-hand-side of Figure 11. We performed the fitting with the help of the Python MDAnalysis Toolkit [55]. The values of the obtained fitting parameters are shown in Table 1, and the values of generalized (or fractional) diffusion coefficient *D_α_* are depicted in Figure 11 (right-hand side). The presented results are very similar to those obtained for the lateral diffusion of DOPC [44]. The results are presented separately for the DPPC (a) and POPE (b), and individually, separately for the bilayer side in contact with B2-GP-1 and the one without contact with the protein (named “noncontact” in Figure 11 and Figure 12). For the DPPC, there was a difference between the contact and noncontact bilayer behavior. The MSD of the contact side had higher values—a bigger diffusion coefficient than the noncontact side. This may indicate that the lipids were pushed into the bilayer by the protein. It comes in a pair with the information of a large number of intermolecular interactions between the phospholipid and protein. Additionally, Lys, Phe, and Thp, in the case of the DPPC, created HP bonds even with the carbons in the tails of the bilayer (see, Supplementary Materials Figure S8)—not only with the heads. Thus, the DPPC’s lipid tail started to be exposed to protein influence, which was not possible in the stable and ordered bilayer. This is in opposition to the POPE, which created a lot of interactions inside the bilayer, so the differences of MSD between the contact and noncontact sides are less visible. The values of *D_α_* were approximately two times greater for the DPPC than for the POPE. This indicates higher diffusivity in the case of DPPC and perhaps the fact that the DPPC was more liquid than POPE. The situation is similar to the one reported in Reference [56], where a comparison of the lateral diffusion coefficient of DPPC with mixture DPPC/DPPE up to pure DPPE bilayer was presented. Also, there are studies in which POPC and POPE discrepancies have shown the greater diffusion coefficient for bilayer with PC head [35]. However, in Reference [57], a comparison of four types of bilayers with PC head and different tails has been presented, and the diffusion coefficient for bilayer with DP tail was also much greater than the rest. Thus, the feature is attributed to both head- and tail-effect, and the presence of the protein only slightly changes this image, which can be observed by discrepancies between contact and non-contact sides.

Another interesting observation is the discrepancy of the fitting parameter, *α,* between the DPPC and POPE bilayers. The values of the parameter for the DPPC, despite being much lower than one in all cases, are significantly lower than for the POPE. This shows that lipid motions are more sub-diffusive in the case of the DPPC (despite the bigger diffusion coefficient). It indicates different dynamics inside each bilayer: the moves inside the DPPC are more confined. No meaningful differences were found when comparing the MD simulation results of the system with the NaCl and the KCl solutions for DPPC. For the POPE, the differences are more visible; however, further research is necessary to discern if such a phenomenon is caused by the type of added ions or randomness.

Based on the fitted MSD functions (see Table 1), the complex viscoelastic moduli, G’ and G’’, were computed using Equation (4) and are presented in Figure 12.

The radius of gyration, Rg, for both bilayers was calculated as an average overall lipid molecule: for the DPPC, *R_g_* = 8.66 Å, and for the POPE, *R_g_* = 8.29 Å. When the molecule moved diffusively, G’’ dominated over G’. When the molecule motion was confined by the elastic structures of the complex system, G’ dominated, which is the case for DPPC (see Figure 12a). The same was true for the POPE, but the discrepancies between G’ and G’’ were smaller, and there were cross points of G’ and G’’ (see Figure 12b). For the DPPC, there was an additional difference in the values of G’ and G’’ for lipid molecules on the contact and noncontact sides. As the POPE was more rigid than the DPPC due to interaction between the heads of the bilayer molecules, the diffusivity and viscoelasticity were different for the two bilayers. The elastic modulus had much smaller values in the case of the DPPC; thus, the bilayer had a smaller resistance to being deformed than the stiffer POPE—the ratio of the viscous to elastic effects was greater in the POPE than the DPPC.

## 4. Conclusions

The phospholipid bilayers covering the articular cartilage are believed to be crucial for the efficient lubrication of a joint [15,20]. The synovial fluid surrounding the bilayers contains a lot of different types of molecules that facilitate the lubrication. The most important components and their role in lubrication have been studied before [20,36,37,46]. β2-GP-1 is one of the proteins found in the synovial fluid, and in this paper, we focused on its influence on the behavior of the lipid bilayers present in the synovial fluid: DPPC and POPE. Our efforts aimed to validate the hypothesis given by theorists and experimentalists (e.g., in Reference [16]) that β2-GP-1 can provoke inactivation of the phospholipid bilayers, which is a cause of the destruction of cartilage in most rheumatic diseases and osteoarthritis. They propose that the inactivation is connected with the detachment of lipid heads (PO_4_^−^) caused by strong interactions between β2-GP-1 and the lipids. We have shown that the binding between the protein and bilayers can take one of two forms: direct (H-bonds, hydrophobic contacts, ionic interactions) and indirect (water bridges). Figure 5 shows that the interaction is mainly hydrophobic. On the other hand, H–bonds are much stronger ~15–30 kJ/mol vs. <2 kJ/mol for HP contacts. While the averaged energy of a single H–bond for DPPC is equal to 19.6 kJ/mol, it is slightly lower for POPE and equals 18.8 kJ/mol. All of the binding interactions are located within a small area (only a few lipids contribute to the binding, with each of them creating few interactions). Even in sum, the contribution of H–bonds and HP interactions to the binding energy is smaller than the covalent bond energy between C and O atoms, which equals about 350 kJ/mol. Thus, the possibility of the intermolecular interactions detaching a lipid’s head cannot be confirmed. However, the proximity of the protein causes water displacement from the vicinity of the bilayer (disrupting water bridges needed for the stabilization of the bilayer), which can contribute to the bilayer’s instability. The protein can push the lipids into the bilayer, which is indicated in DPPC by the HP interactions created between the protein and head-atoms of the lipids and between the protein and tail-atoms.

In the case of DPPC, the protein penetrated the bilayer on the 27–30 Å depth; thus, it penetrates about half the bilayer’s depth. In POPE, the protein penetrates only a shallow of the bilayer, i.e., on the depth equal to 2.5–3.5 Å. Thus, the observation can be found that the head-effect was very important for protein–bilayer interactions. In POPE, the penetration depth was much smaller, and the protein interacted mainly with the heads of the lipids that created a stable barrier. The interactions between POPE heads seem to be too strong to let the protein go inside.

Additionally, the MSD of the lipids can show the pushing effect of the protein by emphasizing the differences in the lipids’ motion on the two lipid layers. The effect is more visible for DPPC than POPE as it is a stronger bond to the protein and penetrated by it; thus, a difference in MSDs between the contact and noncontact bilayer sides can be seen. The MSD of the contact side has higher values—a bigger diffusion coefficient than the noncontact side. Such pushing at a larger scale could disorganize the bilayer. In the case of DPPC, the lipids’ motions were more sub-diffusive and confined than in the POPE case. It also provided bigger discrepancies between the viscous and elastic moduli—the ratio of the viscous-to-elastic effects is greater in POPE than DPPC. POPE created a lot of interactions inside the bilayer, also supported by water bridges that stabilized the bilayer. Thus, the difference between the MSDs between the contact and non-contact sides of the POPE bilayer was much less visible. The secondary structure of the protein did not change much in time in each case.

Summarizing, we have shown how the presence of β2-GP-1 can influence phospholipids’ bilayer behavior, immersed in a water solution with the addition of monovalent ions. We carefully described the interactions inside the system. In future work, we plan to study the influence of divalent ions in the water solution in which the system is immersed and the water behavior near the bilayer, as the importance of such studies has been noticed by other groups [58]. This paper compared the protein binding to the two bilayers (DPPC and POPE), which differ in head- and tail-groups. Thus, we have shown two concurrent effects of the two groups. It would be very interesting to compare the presented results with the two pairs DPPC/POPC and for POPE/DPPE in order to check the influence of the head- and tail-groups on the protein binding separately and this is expected to be carried out in the future research.

However, the real bilayer is somewhat more complex as it is not built from a particular lipid type, but rather several (mixed into one bilayer). Our future work will also address this problem. Moreover, steered molecular dynamics simulations have to be performed to understand the process of bilayer degradation as affected by β2-GP-1. Those would involve using coarse-grained models for better efficiency of calculations for the time scales presented in this work while maintaining the agreement with all-atom models that otherwise could be too computationally costly.

## Figures and Tables

**Table 1:** α parameters based on the fitting function presented in Equation (2). All results were obtained for the bilayers with the presence of the protein bound to it.

Abbreviation	*α* (Contact Side)	STD	*α* (Non-Contact Side)	STD
DPPC + KCl	0.101	0.001	0.150	0.001
DPPC + NaCl	0.105	0.001	0.127	0.001
POPE + KCl	0.383	0.002	0.344	0.002
POPE + NaCl	0.300	0.001	0.244	0.002

## Supplementary Materials

The following are available online at https://www.mdpi.com/2077-0375/10/12/396/s1, Figure S1: Energy of the whole system as a function of time for DPPC (left) and POPE (right) with the presence of the protein bound to it, Figure S2: Histograms of covariances matrix (cf. Equation (4)) of four exemplary-picked atoms of lipid molecule: upper for DPPC, lower for POPE. Left-hand-side graphs show densities for an atom in the head of the lipid, right-hand-side ones show an atom in tail of the lipid. Abbreviations: ck–DPPC+KCl, cna–DPPC+NaCl, ek–POPE+KCl, ena–POPE+NaCl, Figure S3: Time evolution of the number of intramolecular H-bonds inside POPE bilayer with the presence of the protein bound to it. Note, that DPPC has no H-bonds inside(left). Number of intramolecular H-bonds inside POPE bilayer in the absence of protein has been presented for comparison (blue line). The right shows a similar chart for the number of intramolecular H-bonds inside β2-GP-1, Figure S4: Secondary structure as a function of time of the protein bound to the POPE+KCl (125ns of simulations) and the simulation snapshots, Figure S5: Map of β2-GP-1 amino acids H-bonding PL molecules after 50 ns of simulation time: left for DPPC, right for POPE (show which bilayer’s atoms are in contact with the amino acid). Abbreviations: ck–DPPC+KCl, cna–DPPC+NaCl, ek–POPE+KCl, ena–POPE+NaCl, Figure S6: Detailed HP and H-bonds interactions map between the protein and the bilayer: number of amino acids (AA) in sequence connected by one or more HP interaction or H-bond (HBo) and their names (IV domain marked by green, V domain–by yellow). Number of connections marked by blue. The map is created based on only one example of each simulated model system, thus the results can differ from the ones with based on averaged values (eg. sum of intermolecular HP interactions and H-bonds), Figure S7: Number of ionic interactions, coming from ions added in solution, as a function of time. Abbreviations: ck–DPPC+KCl, cna–DPPC+NaCl, ek–POPE+KCl, ena–POPE+NaCl, Figure S8: Ionic interactions as a function of time. All interactions in the system, Figure S9: Time evolution of number of intramolecular HP contacts inside bilayers with the presence of the protein bound to it (left) and numbers of carbon atoms which create these HP interactions in the case of simulations with NaCl solution (right). For a description of the atom numbers, see Figure 2. Number of intramolecular interactions inside DPPC and POPE bilayers in the absence of protein have been presented for comparison (blue and grey lines)., Figure S10: Time evolution of number of intramolecular HP contacts inside β2-GP-1 bound to the bilayer, Figure S11: Map of HP interactions between β2-GP-1 amino acids and phospholipid atoms after 50 ns of simulation time: left for DPPC, right for POPE (show which bilayer’s atoms are in contact with the amino acid–only a part of the atoms has been chosen to show-the ones which were contacted more often). Abbreviations: ck–DPPC+KCl, cna–DPPC+NaCl, ek–POPE+KCl, ena–POPE+NaCl. Figure S12: Structure of DPPC bilayer and a fragment of 2-GP-1 after 50 ns, for 2% NaCl solution of water. Light blue atoms represent carbon, dark blue-nitrogen, red-oxygen, yellow-phosphorus, and white-hydrogen (solvent molecules are hidden). Note that the periodic boundary conditions, present during simulations on each wall, are switched off to show the molecules as non-fragmented (i.e. the simulation space was expanded for presentation purposes). Green lines show the HP interactions between the protein and the bilayer, Figure S13: Electron density profile across DPPC bilayer at the start of the simulation (dashed lines), and the end of the simulation (solid lines). The density of all phospholipids’ atoms (red line), and water atoms (blue line).

## Abbreviations

MD	Molecular Dynamics
β2-GP-1	Beta-2-glycoprotein-1
DPPC	1,2-dipalmitoyl-sn-glycero-3-phosphocholine
POPE	1-palmitoyl-2-oleoyl-sn-glycero-3-phosphoethanolamine
MSD	Mean Squared Displacement
H-bonds	Hydrogen bonds
HP	Hydrophobic-hydrophilic
