# Supplementary material for: Interactions between Beta-2-Glycoprotein-1 and Phospholipid Bilayer—A Molecular Dynamic Study
Source: Membranes (Basel). 2020 Dec 5;10(12):396. doi: 10.3390/membranes10120396 (PMC7762114; doi:10.3390/membranes10120396)
Supplement: Supplementary file 1 [file membranes-10-00396-s001.pdf]

membranes

Supplementary Materials: Interactions between Beta-2-Glycoprotein-1 and Phospholipid Bilayer—A Molecular Dynamic Study

Natalia Kruszewska [1,*], Krzysztof Domino [2], Radosław Drelich [3], Wiesław Urbaniak [3] and Aneta D. Petelska [4,*]

[1] Institute of Mathematics and Physics, UTP University of Science and Technology, Kaliskiego 7, 85-796 Bydgoszcz, Poland
[2] Institute of Theoretical and Applied Informatics, Polish Academy of Sciences, Bałtycka 5, 44-100 Gliwice, Poland; kdomino@iitis.pl
[3] Faculty of Mathematics, Physics and Technical Sciences, Kazimierz Wielki University, Chodkiewicza 30, 85-867 Bydgoszcz, Poland; radeko@ukw.edu.pl (R.D.); wurban@ukw.edu.pl (W.U.)
[4] Faculty of Chemistry, University of Bialystok, Ciolkowskiego 1K, 15-425 Bialystok, Poland
* Correspondence: nkruszewska@utp.edu.pl (N.K.); aneta@uwb.edu.pl (A.D.P.)

Received: 28 October 2020; Accepted: 3 December 2020; Published: date

In Figure S1, the time evolution of the energy of the whole system has been demonstrated. The energy has been evaluated applying the AMBER03 force field (the proper equation has been described in detail in [44]). In each case, a similar decreasing tendency can be seen. In the case of DPPC, the energy decreases for about 2·104 kJ/mol, but in POPE, it is about 1·104 kJ/mol. The figures also show how the addition of ions into solvent changes the energetic picture of the system. In the presented simulation, as usually in molecular dynamic simulations, the ions have been added to the solution to neutralize the system at a physiological ion concentration. In the case of KCl, the energies are higher than in the case of NaCl (for DPPC 2.8% higher and POPE 2.9% higher). In all cases, the stabilization of the system energy can be seen at the simulation time of around 40-50 ns.

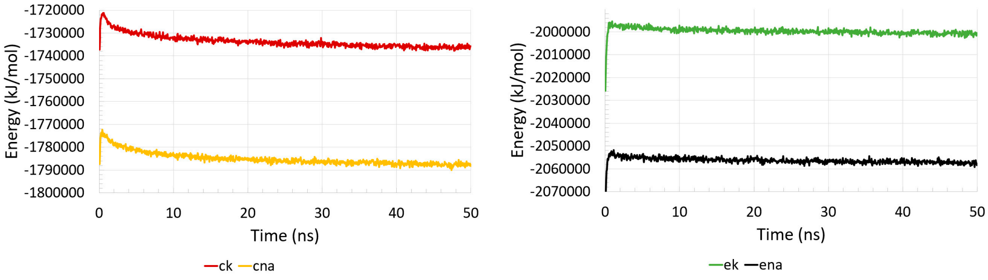

Figure S1. Energy of the whole system as a function of time for DPPC (left) and POPE (right) with the presence of the protein bound to it. Abbreviations: ck – DPPC+KCl, cna – DPPC+NaCl, ek – POPE+KCl, ena – POPE+NaCl.

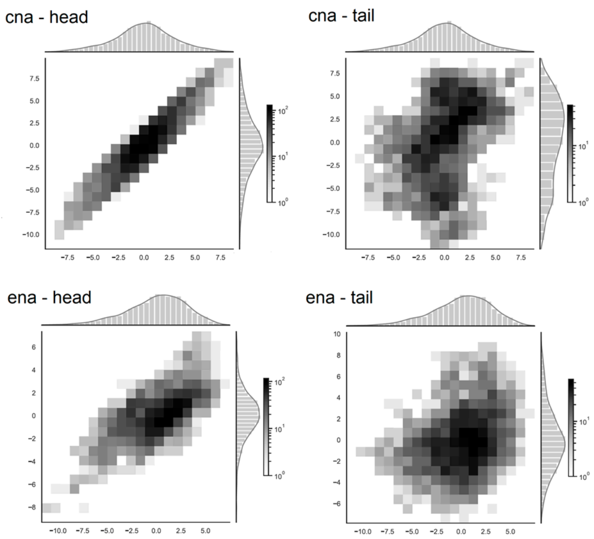

Figure S2. Histograms of covariances matrix (cf. Equation (4)) of four exemplary-picked atoms of lipid molecule: upper for DPPC, lower for POPE. Left-hand-side graphs show densities for an atom in the head of the lipid, right-hand-side ones show an atom in tail of the lipid. Abbreviations: ck – DPPC+KCl, cna – DPPC+NaCl, ek – POPE+KCl, ena – POPE+NaCl.

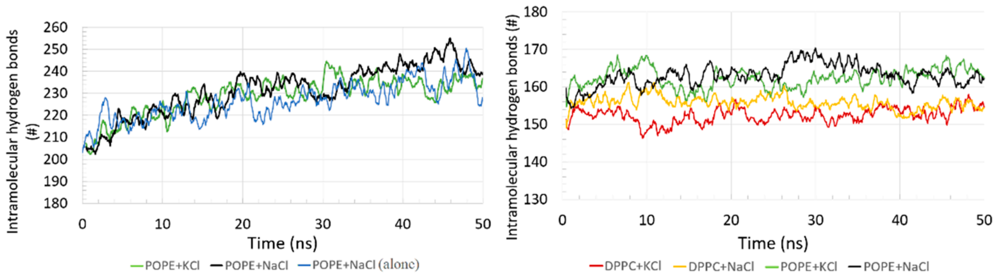

Figure S3. Time evolution of the number of intramolecular H-bonds inside POPE bilayer with the presence of the protein bound to it. Note, that DPPC has no H-bonds inside(left). Number of intramolecular H-bonds inside POPE bilayer in the absence of protein has been presented for comparison (blue line). The right shows a similar chart for the number of intramolecular H-bonds inside β2-GP-1.

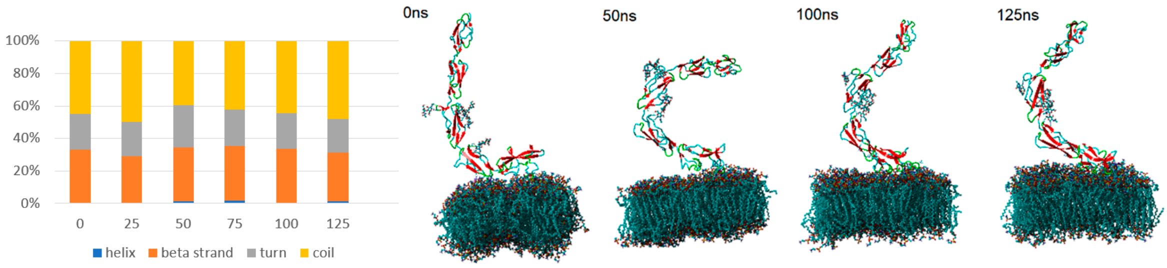

Figure S4. Secondary structure as a function of time of the protein bound to the POPE+KCl (125ns of simulations) and the simulation snapshots.

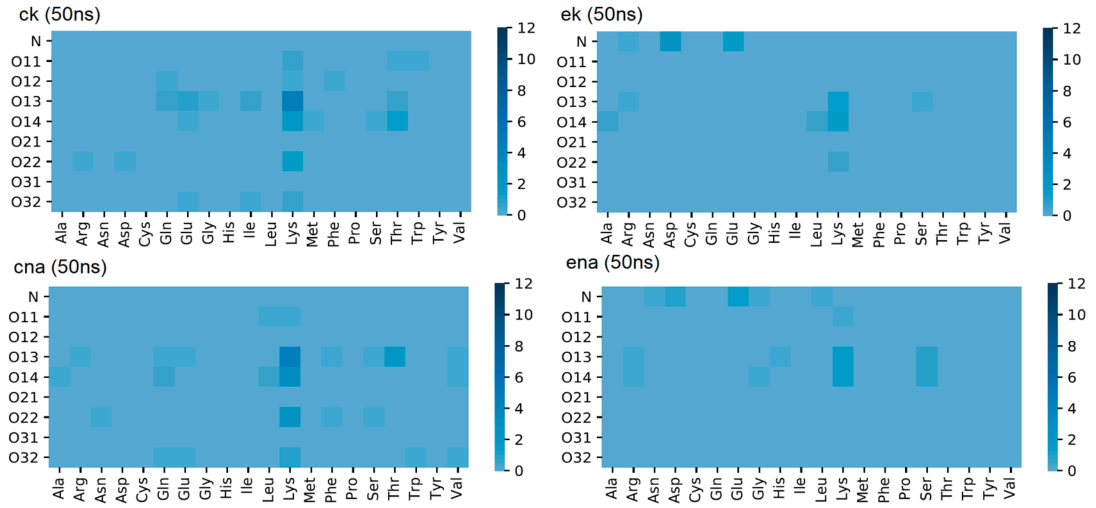

Figure S5. Map of β2-GP-1 amino acids H-bonding PL molecules after 50 ns of simulation time: left for DPPC, right for POPE (show which bilayer's atoms are in contact with the amino acid). Abbreviations: ck – DPPC+KCl, cna – DPPC+NaCl, ek – POPE+KCl, ena – POPE+NaCl.

Intermolecular HP interactions	DPPC+NaCl	Number	2	2	7	2	3	7	7	2	3	1	2	4	1	7	8	1	1	3	2	1	1	1	2	1	1	5	4	3	2	1	1	4	1	6	7	2
		AA name	219 TYR	244 SER	246 LYS	247 LEU	249 VAL	250 LYS	251 LYS	254 VAL	256 TYR	262 LYS	263 ILE	264 GLN	266 LYS	267 PHE	268 LYS	270 GLY	272 LEU	277 VAL	279 PHE	292 GLU	294 ALA	300 THR	303 VAL	304 PRO	305 LYS	307 PHE	308 LYS	309 GLU	310 HIS	311 SER	312 SER	313 LEU	314 ALA	315 PHE	316 TRP	318 THR
	DPPC+KCl	Number	1	1	3	2	1	7	2	2	1	1	1	1	1	6	6	2	2	1	2	2	3	2	6	4	1	2	4	11	5	26	10	1	1			
		AA name	219 TYR	244 SER	246 LYS	247 LEU	248 PRO	250 LYS	251 LYS	252 ALA	261 VAL	262 LYS	263 ILE	265 GLU	266 LYS	267 PHE	268 LYS	269 ASN	279 PHE	300 THR	301 ILE	302 GLU	303 VAL	305 LYS	307 PHE	308 LYS	309 GLU	310 HIS	311 SER	313 LEU	314 ALA	315 PHE	316 TRP	317 LYS	318 THR			
	POPE+KCl	Number	1	1	1	1	1	3	3	8																												
		AA name	218 GLY	219 TYR	284 LYS	311 SER	312 SER	313 LEU	314 ALA	315 PHE																												
	POPE+NaCl	Number	1	1	1	2	4																															
		AA name	219 TYR	250 LYS	300 THR	312 SER	313 LEU																															
Intermolecular HBo	DPPC+NaCl	Number	2	1	1	3	1	2	1	2	1	1	1	1																								
		AA name	246 LYS	250 LYS	251 LYS	262 LYS	264 GLN	266 LYS	267 PHE	268 LYS	305 LYS	308 LYS	313 LEU	318 THR																								
	DPPC+KCl	Number	1	2	1	1	1	1	1	1	1	1	3	1	2	1																						
		AA name	246 LYS	250 LYS	251 LYS	265 GLU	266 LYS	267 PHE	268 LYS	270 GLY	301 ILE	305 LYS	308 LYS	317 LYS	318 THR	319 ASP																						
	POPE+KCl	Number	1	1	1	1	2	1	2	2	1	1																										
		AA name	193 ASP	194 ASN	217 ASP	218 GLY	246 LYS	284 LYS	303 VAL	308 LYS	313 LEU	314 ALA																										
	POPE+NaCl	Number	1	1	1	3	1	1																														
		AA name	193 ASP	194 ASN	302 GLU	305 LYS	311 SER	312 SER																														

Figure S6. Detailed HP and H-bonds interactions map between the protein and the bilayer: number of amino acids (AA) in sequence connected by one or more HP interaction or H-bond (HBo) and their names (IV domain marked by green, V domain – by yellow). Number of connections marked by blue. The map is created based on only one example of each simulated model system, thus the results can differ from the ones with based on averaged values (eg. sum of intermolecular HP interactions and H-bonds).

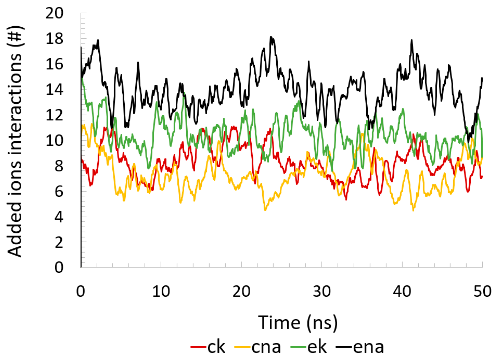

Figure S7. Number of ionic interactions, coming from ions added in solution, as a function of time. Abbreviations: ck – DPPC+KCl, cna – DPPC+NaCl, ek – POPE+KCl, ena – POPE+NaCl.

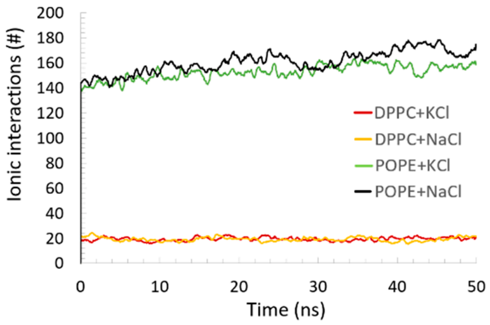

Figure S8. Ionic interactions as a function of time. All interactions in the system.

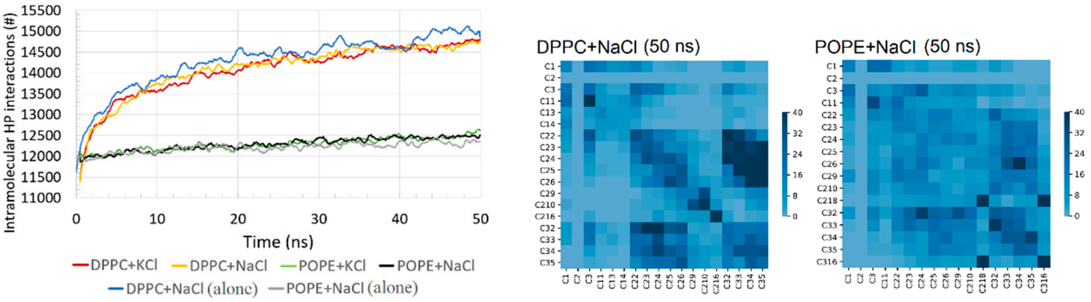

Figure S9. Time evolution of number of intramolecular HP contacts inside bilayers with the presence of the protein bound to it (left) and numbers of carbon atoms which create these HP interactions in the case of simulations with NaCl solution (right). For a description of the atom numbers, see Figure 2. Number of intramolecular interactions inside DPPC and POPE bilayers in the absence of protein have been presented for comparison (blue and grey lines).

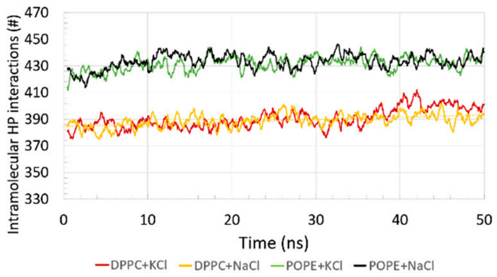

Figure S10. Time evolution of number of intramolecular HP contacts inside β2-GP-1 bound to the bilayer.

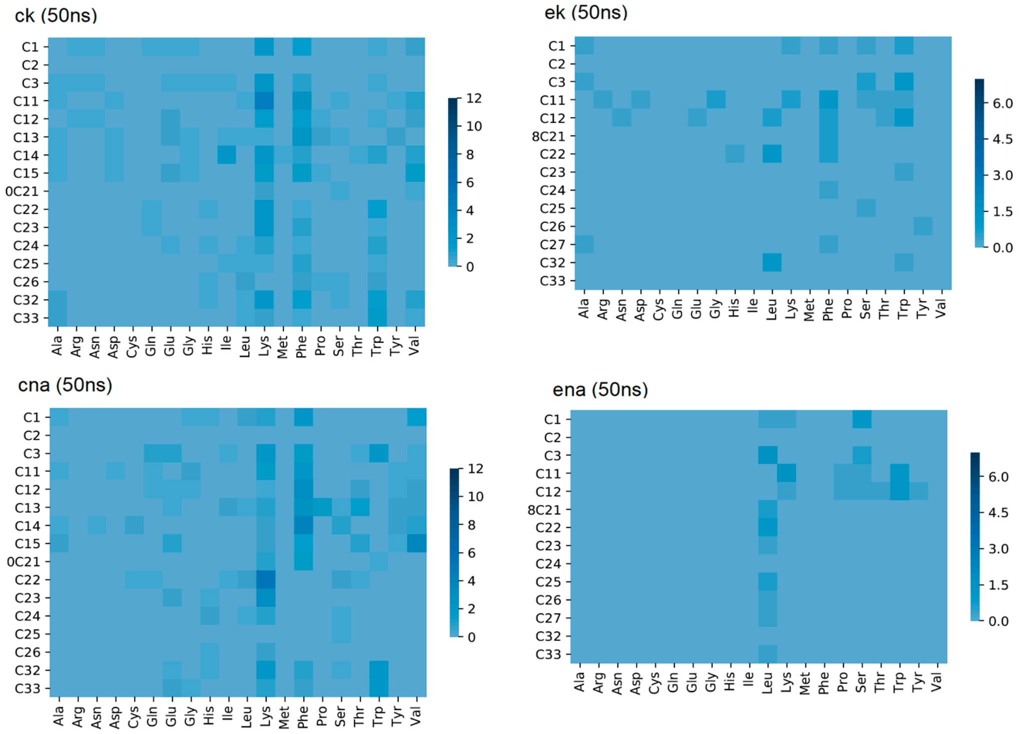

Figure S11. Map of HP interactions between β2-GP-1 amino acids and phospholipid atoms after 50 ns of simulation time: left for DPPC, right for POPE (show which bilayer's atoms are in contact with the amino acid – only a part of the atoms has been chosen to show - the ones which were contacted more often). Abbreviations: ck – DPPC+KCl, cna – DPPC+NaCl, ek – POPE+KCl, ena – POPE+NaCl.

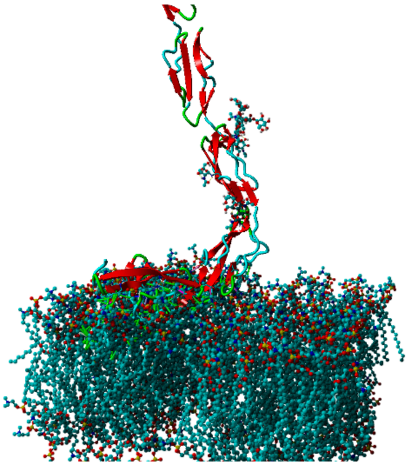

Figure S12. Structure of DPPC bilayer and a fragment of 2-GP-1 after 50 ns, for 2% NaCl solution of water. Light blue atoms represent carbon, dark blue - nitrogen, red - oxygen, yellow - phosphorus, and white - hydrogen (solvent molecules are hidden). Note that the periodic boundary conditions, present during simulations on each wall, are switched off to show the molecules as non-fragmented (i.e. the simulation space was expanded for presentation purposes). Green lines show the HP interactions between the protein and the bilayer.

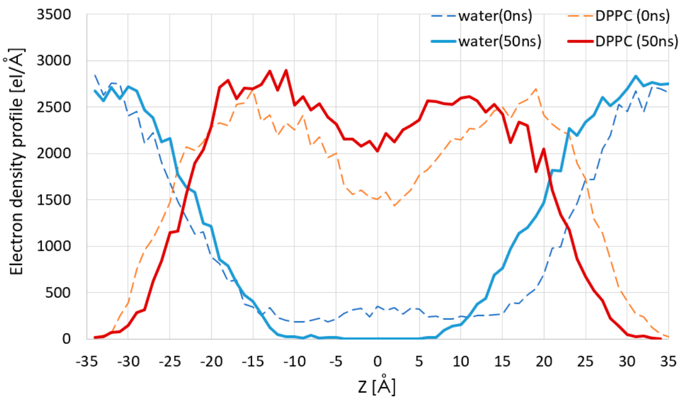

Figure S13. Electron density profile across DPPC bilayer at the start of the simulation (dashed lines), and the end of the simulation (solid lines). The density of all phospholipids' atoms (red line), and water atoms (blue line).

Publisher's Note: MDPI stays neutral with regard to jurisdictional claims in published maps and institutional affiliations.

© 2020 by the authors. Submitted for possible open access publication under the terms and conditions of the Creative Commons Attribution (CC BY) license (http://creativecommons.org/licenses/by/4.0/).
